# The PSO-IFAH optimization algorithm for transient electromagnetic inversion

**DOI:** 10.1371/journal.pone.0317596

**Published:** 2025-01-22

**Authors:** Zhengyu Xu, Guofeng Zhao, Xian Liao, Nengyi Fu

**Affiliations:** 1 School of Electrical Engineering, Chongqing University, Chongqing, China; 2 State Key Laboratory of Power Transmission Equipment Technology, School of Electrical Engineering, Chongqing University, Chongqing, China; 3 Geological Exploration Technology Institute of Jiangsu Province, Nanjing, China; 4 Jiangsu Province Engineering Research Center of Airborne Detecting and Intelligent Perceptive Technology, Nanjing, China; 5 Department of Electrical and Computer Engineering, The University of Tulsa, Tulsa, OK, United States of America; Vellore Institute of Technology, INDIA

## Abstract

As a non-contact method, the transient electromagnetic (TEM) method has the characteristics of high efficiency, small impact of device, no limitation of site range, and high resolution, and is a hot topic in current research. However, the research on the refined data processing method of TEM is lag, which seriously restricts the application in superficial engineering investigation and is a key problem that needs to be solved urgently. The particle swarm optimization (PSO) algorithm and firefly algorithm (FA) were successful swarm intelligence algorithms inspired by nature. However, the accuracy and efficiency of the algorithm restrict its further development. In this paper, the particle moving velocity of FA algorithm is defined according to the concept of particle moving velocity in PSO algorithm, so as to improve the local fast convergence ability of FA algorithm. On this basis, the appropriate velocity of particle movement is improved, so that the improved algorithm can overcome the oscillation problem around the optimal solution and improve the computational efficiency. And finally, an improved PSO-IFA hybrid optimization algorithm (PSO-IFAH) was proposed in the paper. The proposed algorithm can exploit the strong points of both PSO and FA algorithm mechanisms. A typical layered model was established, and the PSO algorithm, FA algorithm, and PSO-IFAH algorithm were applied to inversion calculations. The results show that the PSO-IFAH algorithm improves calculation accuracy by more than 80% and efficiency by over 60% compared to the PSO and FA algorithms, respectively. The PSO-IFAH algorithm also exhibits high inversion accuracy and stability, with superior anti-noise properties compared to the other algorithms. When implemented in ground TEM measurement data processing, the PSO-IFAH algorithm enhances the resolution of anomalies and low-resistance details, aligning well with actual excavation results. This highlights the algorithm’s capability to depict underground electrical structures and karst developments accurately, thereby improving the precision of TEM data processing and interpretation.

## 1. Introduction

**Fig 1:**
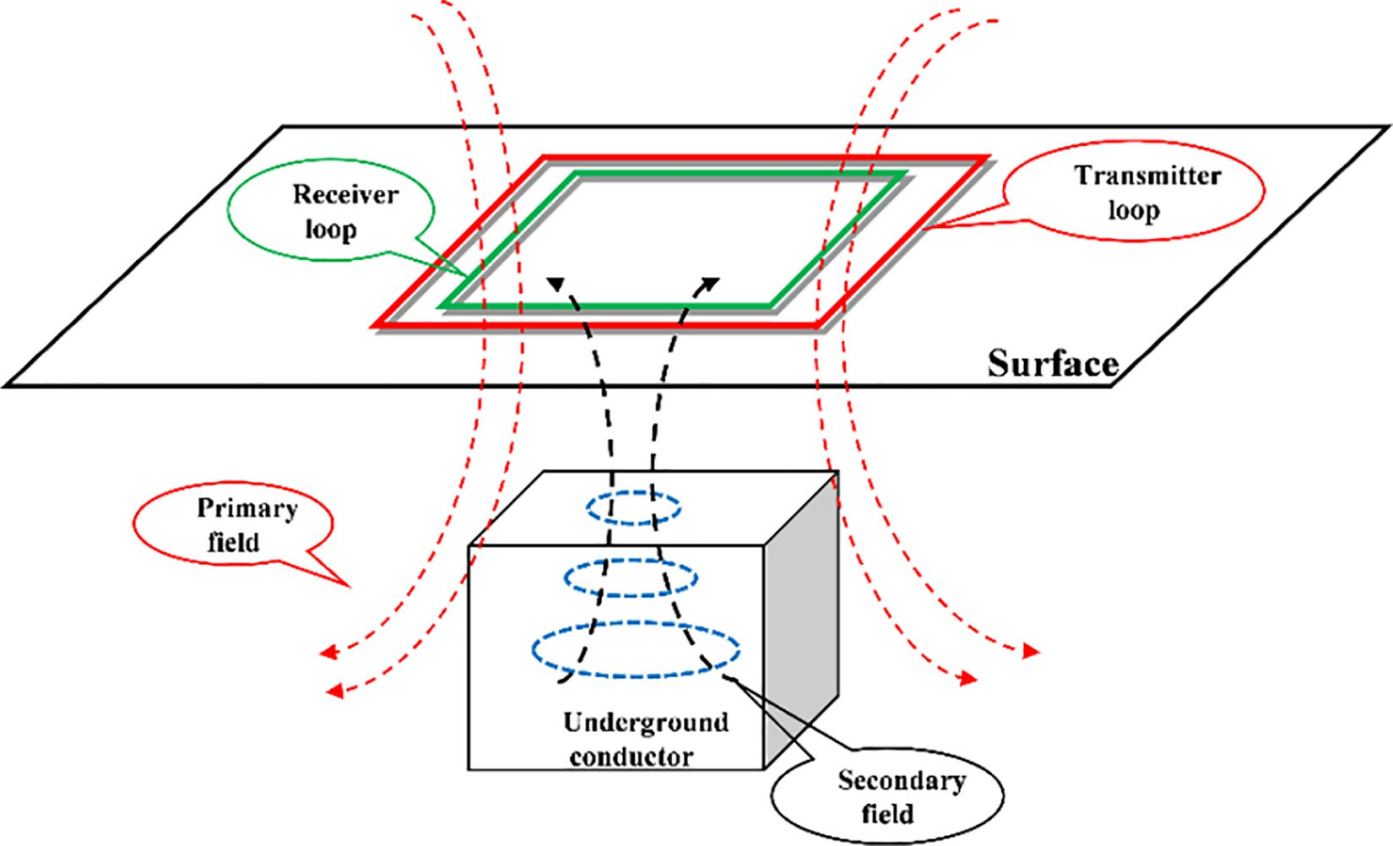
Schematic diagram of the transient electromagnetic method.

The transient electromagnetic (TEM) method is a type of time-domain electromagnetic detection method for studying the resistivity distribution of the earth’s surface and the working schematic diagram of this method is shown in Fig 1. The primary fields were generated by the transmitter current into the red transmitter loop which was on the surface. During the transmitter current was turned off, the secondary fields were induced by a conductor medium within the earth and observed by using a green receiving loop. The distribution of conducting medium inside the earth can be determined by processing and analyzing the secondary fields [1, 2]. In practical work, the TEM methods can solve mineral resource exploration, coal mine water and mud inrush investigation, and karst investigation by observing the changes in the secondary fields over time [3, 4].

It is a complicated process to convert the TEM secondary fields into the resistivity information of the earth’s stratum by mathematical methods [5–7]. The smoke-ring fast imaging method was adopted by early geophysical researchers [8–10]. Some linear iterative methods have also been used in geophysics inversion calculations, such as the Gauss-Newton method, Damped Least Square method, Adaptive regularization inversion method, and so on [11–13]. The advantages of the above methods are fast convergence and relatively small calculation. At the same time, there are also the following shortcomings. For example, the smoke-ring fast imaging method was approximate and rough and had low data processing accuracy. In the inversion process, the linear iterative method has the following shortcomings: it is highly dependent on the initial model, the inversion result is easy to fall into a local minimum, and the sensitivity matrix has a heavy computing amount and high requirements for computer processors. Hence, geophysical researchers chose to apply nonlinear optimization algorithms to geophysical inversion and achieved success [14, 15]. The nonlinear optimization algorithms can easily jump out of local minima and converge to a global minimum in the searching process and have been widely used in geophysical inversion in recent years [16–18]. For example, Li and Boschetti *et al*. used a whale and genetic algorithm for potential field data inversion [19, 20]; Roy and Bosch *et al*. applied the Monte Carlo algorithm to gravitational field data and magnetic field data inversion [21, 22], and Yin *et al*. applied the simulated annealing algorithm to airborne electromagnetic data inversion [23]. The Particle Swarm Optimization (PSO) algorithm was first proposed by Kennedy and Eberhart in 1995, and has shown good performance in terms of global search ability and convergence speed, and has been successfully used to solve problems in many fields [24, 25]. Fernández *et al*. first applied the PSO algorithm to electrical-sounding data inversion [26]. Godio and Yuan *et al*. successfully processed magnetotelluric data and other geophysical data using the PSO algorithm [27, 28]. In TEM inversion, Xu *et al* applied the PSO algorithm for TEM data inversion and the results show that the algorithm has the advantage of high efficiency [29]. However, the above research content does not consider the influence of random noise on the accuracy of the PSO algorithm.

In 2008, Yang proposed a new algorithm, the Firefly Algorithm (FA), which is an interdisciplinary intelligent and random algorithm with powerful functions and has been successfully applied in many fields [30–32]. The core idea of the algorithm comes from the foraging and courtship behaviors of fireflies using the biological characteristics of light emission. Since the FA algorithm was proposed, it has received the attention and research of many researchers and has been continuously improved. Abdullah proposed a hybrid evolutionary firefly algorithm [33]; Gao presented a dynamic population firefly algorithm based on chaos theory [34]; Xie put forward a firefly algorithm based on chaos optimization and VFSA algorithm [35]; Wang proposed A firefly algorithm based on the logarithmic decrease of inertial weight [36]; Hao came up with a firefly algorithm with adaptive step size [37]; The algorithm has advantages of good search capabilities and optimization capabilities, easy operation and strong practicability, few parameters, and is widely used in engineering, finance, and other fields. However, in the field of geophysical inversion, the development of FA methods is very slow. At present, only Zhou *et al*. first introduced the FA algorithm to Rayleigh wave data inversion [38]; Wang *et al*. performed this method for the data inversion of Magnetotelluric sounding [39]. For transient electromagnetic data inversion, Xu proposes to apply the firefly algorithm to the transient electromagnetic data inversion and succeeds, and on this basis, the application effects of the particle swarm optimization algorithm and firefly algorithm in transient electromagnetic inversion are compared and analyzed [40].

In the research of TEM nonlinear inversion, the PSO algorithm is widely used, but the method has the problem of precocity and results in low calculation accuracy [41]. FA algorithm is an emerging optimization algorithm, but there are problems such as low computation efficiency [40]. In view of the problems existing in the above two algorithms, this paper conducts research. Firstly, the problems of the above two algorithms are analyzed, and an improved PSO-IFA hybrid optimization algorithm (PSO-IFAH) is proposed on this basis. Compared with the PSO algorithm and FA algorithm, the calculation efficiency of the PSO-IFAH optimization algorithm is increased by 65.22%, and the accuracy is improved by 87.62%. A typical layered model was established, and the "smoke ring" fast imaging method, Occam linear iterative method, PSO algorithm, FA algorithm, and PSO-IFAH algorithm were applied to TEM inversion, and the influence of the anti-noise ability was analyzed. On this basis, the improved PSO-IFAH algorithm is applied to the inversion of the measured data, and the inversion results are compared and analyzed with the known geological data. The research content provides a new and effective method for the fine data processing and interpretation of TEM.

## 2. Principles of nonlinear optimization methods

### 2.1 Improved PSO-IFAH optimization algorithm

The particle swarm optimization (PSO) algorithm is jointly proposed based on a model that describes birds or fish looking for food. In an optimization solution, a bird or fish is simplified into particles. In recent years, as a new swarm intelligence-based algorithm that makes it possible to solve complex optimization problems, it has become one of the most important algorithms and has attracted more and more attention. The special information transfer mechanism between particles helps to solve different problems at a lower computational cost and has good performance. The algorithm has the advantages of simple implementation, minimal mathematical processing, and good optimization ability, and it requires less computing memory and is easy to implement. It is found that PSO is robust and fast for nonlinear, non-differentiable, and multimodal optimization problems.

However, the PSO algorithm also has some disadvantages. When implementing the variance of the algorithm, it is necessary to get rid of local optimal to avoid premature convergence. If the number of variables to be solved increases, the optimization problem becomes more complex, so the probability of the algorithm finding a global optimal solution decreases.

In the PSO algorithm, the position of the particle movement represents the possible solution to the problem being solved, while the position of the food represents the optimal solution to the problem being solved. Under certain movement rules, all particles move towards the location of the food, that is, the direction of the optimal solution. Correspondingly, in geophysical inversion, the distance between particles and food is the objective function constructed in the inversion problem [29].

**Fig 2:**
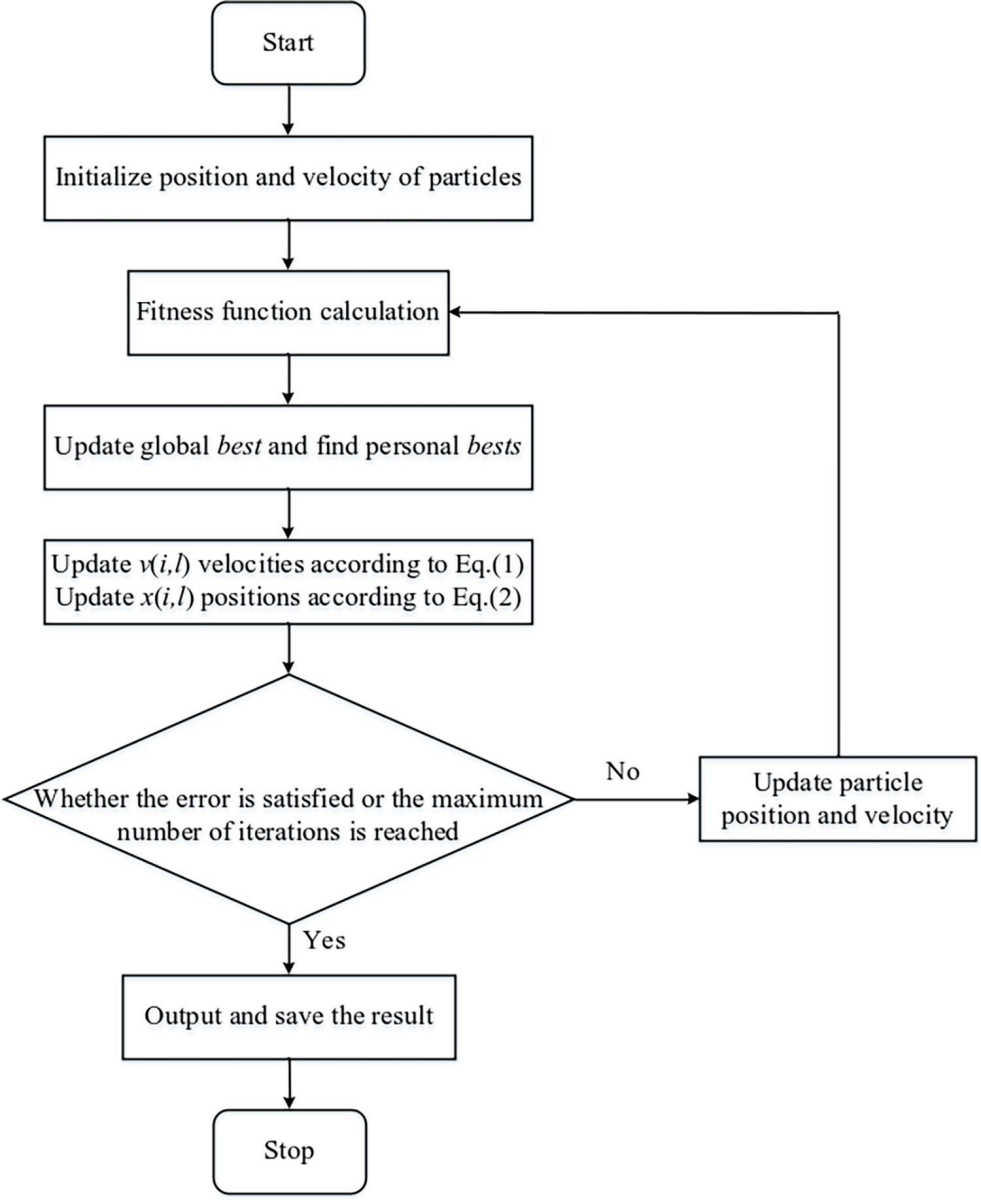
Particle swarm optimization algorithm flowchart.

The Fig 2 shows the flowchart of the particle swarm optimization algorithm. At the beginning of the algorithm, m particles are randomly set as the initial iteration value in the optimal solution search space. Each particle updates its speed and position in real-time according to the equation, and the formula is as follows [26]:

$$v_{i,l}^{k+1}=\omega v_{i,l}^{k}+c_{1}r_{1}(p_{i,l}^{k}-x_{i,l}^{k})+c_{2}r_{2}(g_{i,l}^{k}-x_{i,l}^{k})$$ (1)

$$x_{i,l}^{k+1}=x_{i,l}^{k}+v_{i,l}^{k+1}$$ (2)

Where *i* represents the particle number; *l* represents the dimension of the search space; *k* represents the number of iterations; *c*_1_ and *c*_2_ are learning factors; *r*_1_ and *r*_2_ are two random numbers evenly distributed between (0,1); $p_{i,l}^{k}$ is the optimal position of the individual; $g_{i,l}^{k}$ optimal position for the group; $v_{i,l}^{k+1}$ and $x_{i,l}^{k+1}$ is the speed and position of the *i* particle at *k+1* iteration; *ω* represents the inertia factor and the following formula is:

$$\omega (k)=0.99^{k}•rand/2+a$$ (3)

Where *α* represents a constant and usually between 0~0.5; *rand* represents a random number evenly distributed between (0,1); *k* represents the number of iterations.

The firefly algorithm (FA) is a heuristic algorithm based on the luminous and luminous characteristics of fireflies. In the optimization solution, each firefly position represents the possible solution of the problem to be solved, and the luminous brightness of the firefly represents the objective function value of the problem to be solved. A higher brightness indicates a better objective function value. In the population, fireflies with weak brightness are constantly attracted by the bright and move towards it. As iterative calculations progress, the weakly brightened fireflies in the population are constantly moving towards fireflies that are brighter than themselves. Eventually, most fireflies gather near the brightest fireflies, i.e. the brightest firefly location is the optimal solution to the problem to be solved [30, 31].

**Fig 3:**
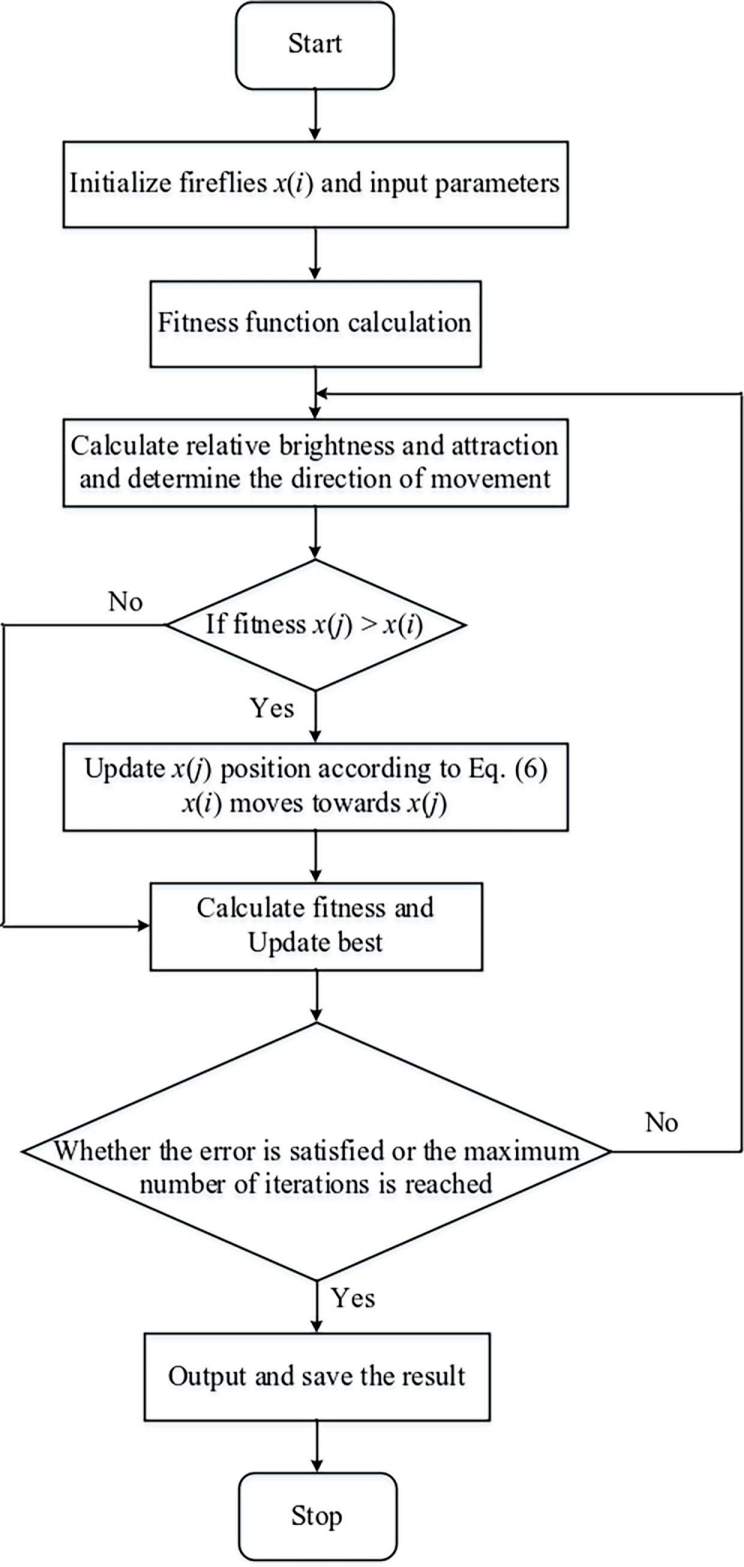
Firefly optimization algorithm flowchart.

The Fig 3 shows the flowchart of the firefly optimization algorithm. In the firefly algorithm, there are two variables, brightness, and attraction degree, and brightness is divided into absolute brightness and relative brightness. Relative to firefly *i*, the initial brightness (brightness at *r = 0*) is called absolute brightness and is denoted as *I*_*0*_; The brightness of firefly *i* at the location of firefly *j*is called relative brightness, denoted as *I* and the formula is as follows [30]:

$$I=I_{0}\times e^{-\gamma r_{ij}}$$ (4)

where *γ* is expressed as the light absorption coefficient; *r*_*ij* is_ expressed as the distance between firefly *i* and firefly *j*; The absolute brightness is related to the objective function, and the better the objective function value, the greater the brightness. In the inversion calculation, the reciprocal of the objective function represents absolute brightness in this paper.

The attraction degree is calculated as follows [30]:

$$\beta_{ij}=\beta_{0}\exp(-\gamma r_{ij}^{2})$$ (5)

Where *β*_*0*_ is the maximum attraction and usually 1 is taken. When firefly *j* moves towards firefly *i*, the position update formula for firefly *j* is [30]:

$$x_{j}^{k+1}=x_{j}^{k}+\beta_{ij}(x_{i}^{k}-x_{j}^{k})+\alpha \in_{i}$$ (6)

Where ∈_*i*_ is the vector of random variables and Randomization parameter(*α*)∈[0, 1].

Xu et al. have analyzed and compared the application effects of the PSO algorithm and FA algorithm in TEM inversion, and the results show that the calculation accuracy of the FA algorithm is better than that of the PSO algorithm, and the computational efficiency of the PSO algorithm is higher than that of FA algorithm [41].

In nonlinear optimization algorithms, the convergence speed is important in the early stage of algorithm iteration. The particle swarm optimization algorithm has a faster convergence ability than other algorithms on some problems. However, in the local search stage, the fast convergence ability of the PSO decreases, especially when searching in the solution space close to the global optimal solution. The magnitude of the particle velocity value is important in a local search, but calculating the appropriate velocity in a local search can be a challenging task because an inappropriate velocity value can cause the particle to oscillate around the optimal solution like a pendulum. These oscillating or oscillating motions cause some delay throughout the optimization task. On the other hand, the Firefly algorithm doesn’t have a speed characteristic. Yang pointed out the superiority of the firefly algorithm over the particle swarm optimization algorithm and the genetic algorithm, and in general, it is easy to achieve global optimization in the optimization problem. In addition, there are no parameters in the firefly algorithm to hold the previous best position of each firefly [31]. Fireflies move regardless of their previous optimal position. Researchers need to improve the PSO algorithm and FA algorithm and put forward a variety of improved optimization algorithms. A hybrid FAPSO algorithm is proposed in reference [42]. When the algorithm is executed, it needs to classify the population and divide the search space into countless small computational subregions for computational search. Reference [43] uses the PSO algorithm and FA algorithm to solve data transmission and transmission in wireless sensors. A hybrid FFPSO algorithm is proposed in reference [44]. FFPSO algorithm and FA algorithm compute the same steps, the difference is that the particle moving position vector is modified. Reference [45] improved the particle moving speed in the FA algorithm to enhance the ability of the algorithm to jump out of the local minimum value.

Therefore, in view of the problems existing in the above two algorithms, combined with the characteristics of the two algorithms, an improved PSO-IFA hybrid optimization algorithm (PSO-IFAH) is proposed. Compared with the particle swarm optimization algorithm, the firefly algorithm doesn’t have the concept of velocity and individual optimal position (pbest). Due to the fast convergence speed of particle swarm optimization in the search process, the PSO algorithm is generally used for global search. In addition, the FA algorithm is often used for local search because it has the function of jumping out of local convergence.

Firstly, the initial calculation parameters of the PSO algorithms and FA algorithms are inputted; Secondly, the optimal position of the individual and the optimal position of the population are randomly calculated within the initial model search range, and in the next comparison stage, the fitness value of the particles in the last iteration is compared according to Eq (7).


$$f(i,k)=\{\begin{array}{l} yes,if\;fitness(x_{i}^{k})\leq g_{i,l}^{k}\\ no,if\;fitness(x_{i}^{k})>g_{i,l}^{k} \end{array}$$ (7)

If the current optimal solution is less than the previous optimal solution, the current optimal solution is saved and recorded, and the FA algorithm is used to perform the next local search. To obtain a suitable moving speed, Eq (6) needs to be further improved. Because improper velocity values can cause particles to oscillate around the optimal solution like a pendulum, these oscillations or oscillations cause some delay throughout the optimization task, increasing the algorithm’s computation time. The current position is then saved in a temp variable *x*_np_, and the new position and velocity of the particle are calculated according to Eqs (8) and (9).

$$x_{j}^{k+1}=x_{j}^{k}+\beta_{ij}(x_{i}^{k}-g_{i,l}^{k})+\alpha (rand-1/2)\varepsilon_{j}$$ (8)

$$v_{i,l}^{k+1}=x_{j}^{k+1}-x_{np}$$ (9)

Where *k* and *α* represent the number of iterations and the step factor; *ε*_*j*_ represents the randomly generated position vector within the parameter range, which is helpful to increase the search area and jump out of the local optimal solution.

**Fig 4:**
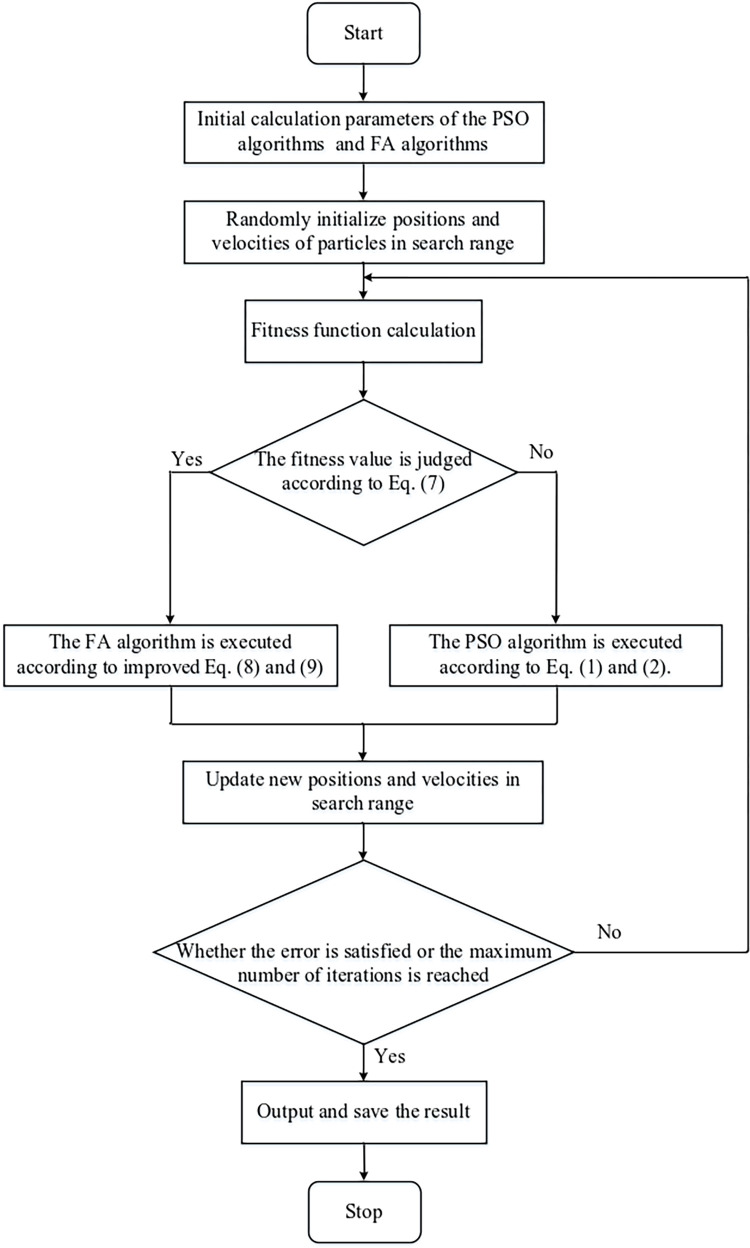
Improved PSO-IFA hybrid optimization algorithm flowchart.

If the current optimal solution is greater than the previous optimal solution, it doesn’t need to be saved. The next iteration calculation is performed according to the particle swarm iteration calculation Formulas (1) and (2). Each iteration of the calculation needs to be judged until the algorithm reaches the maximum number of iterations or error accuracy, and the output saves the optimal solution. The flow diagram of the improved PSO-IFA hybrid optimization algorithm is shown in Fig 4.

### 2.2 Algorithm test

To verify the convergence effect of the improved PSO-IFAH optimization algorithm, the Aekley function is used for testing, and the formula is as follows [41]:

$$f(x)=20\cdot e^{\left(-0.2\sqrt{\frac{1}{2}\sum_{i=1}^{2}x_{i}^{2}}\right)}+e^{\left[\frac{1}{2}\sum_{i=1}^{2}\cos(2\pi x_{i})\right]}-20-e\;x_{i}\in [-5.12,+5.12]$$ (10)

**Fig 5:**
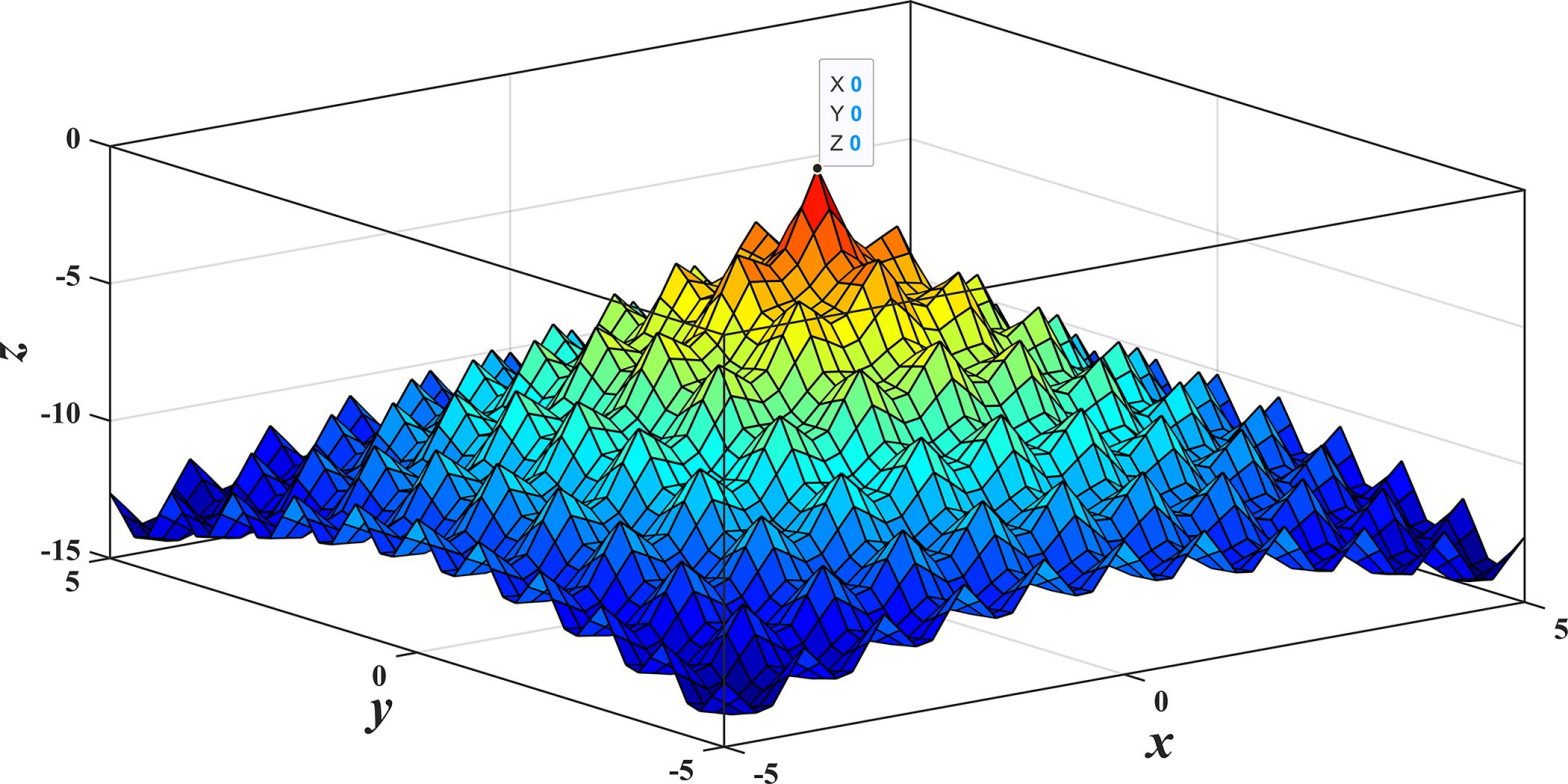
The image of the Aekley function.

Fig 5 shows an image of Aekley’s function. As can be seen from the figure, the Aekley function has a unique global extremum point in the computational domain. Therefore, this function can be used to test the global search capability of the optimization algorithm.

**Fig 6:**
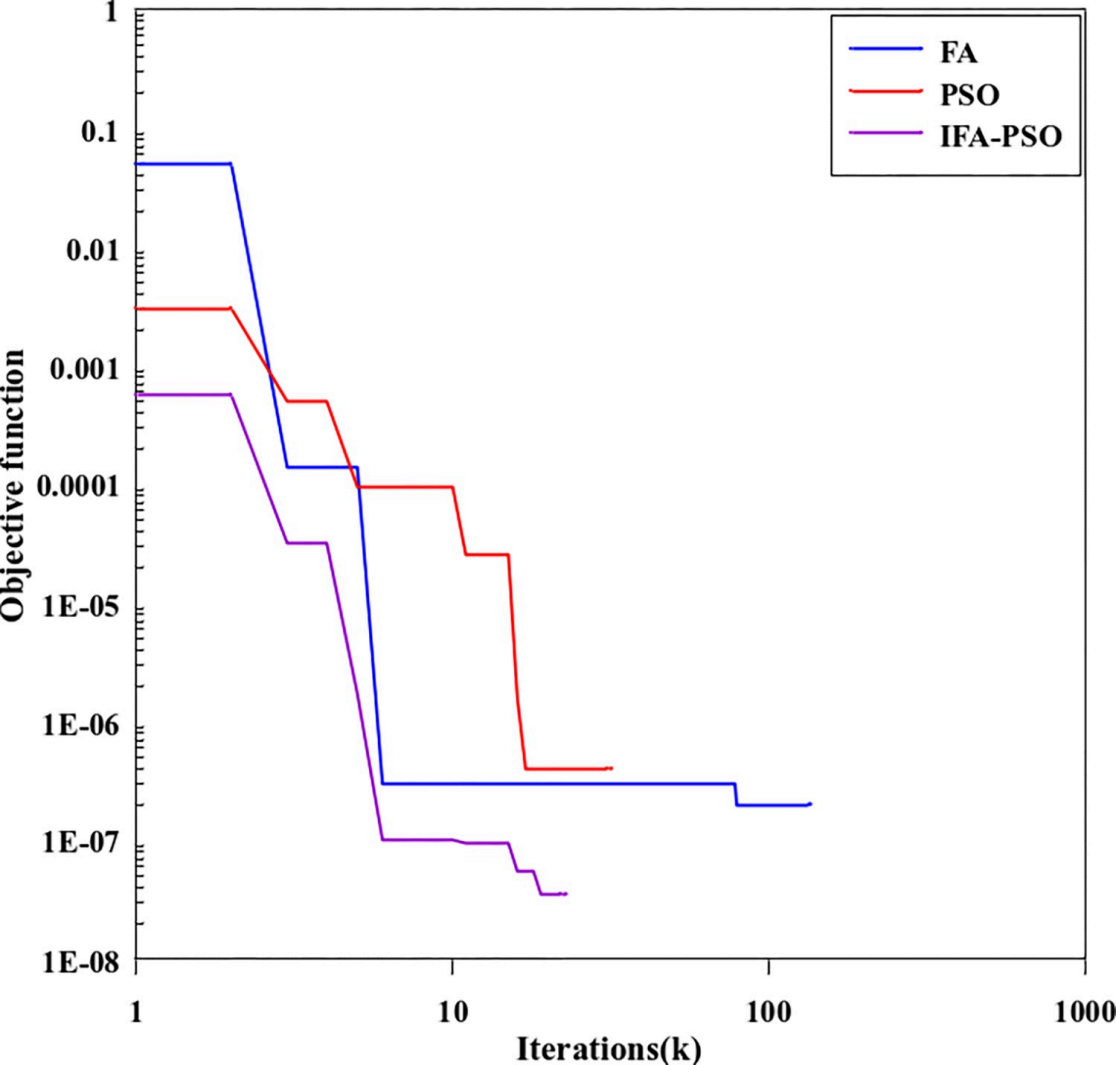
The contrast curve of the algorithm convergence characteristics.

Fig 6 shows the comparison curve of convergence characteristics of the PSO algorithm, FA algorithm, and PSO-IFAH algorithm. As can be seen from Fig 3, the improved PSO-IFAH algorithm shows better characteristics in convergence speed and calculation accuracy after combining the characteristics of the FA algorithm and PSO algorithm. The PSO algorithm converges to the global minimum for 36 iterations, which takes 0.912min. The FA algorithm converges to the global minimum for 161 iterations, which takes 4.561min. The PSO-IFAH algorithm converges to the global minimum for 56 iterations, which takes 1.586min. In terms of computational efficiency, compared with the FA algorithm, the computational efficiency of the PSO-IFAH optimization algorithm is increased by 65.22%. According to the iterative error curve analysis in the figure, compared with the PSO algorithm, the calculation accuracy of the PSO-IFAH optimization algorithm is improved by 87.62%. The convergence speed of the improved algorithm is faster, the error of the objective function is smaller, and the calculation accuracy is improved. Therefore, the results show that the improved PSO-IFAH algorithm has a better global convergence effect and higher computational efficiency.

## 3. Inversion of typical layered models

In the above content, the advantages and disadvantages of the PSO algorithm and FA algorithm are analyzed. On this basis, the PSO-IFAH optimization algorithm with a good convergence effect is proposed. Next, a typical stratified geoelectric model is established for inversion calculation. The PSO algorithm, FA algorithm, and improved PSO-IFAH optimization algorithm are used for inversion calculation, and the calculation accuracy and efficiency of the PSO algorithm, FA algorithm, and improved PSO-IFAH optimization algorithm are discussed. In this paper, when a nonlinear optimization algorithm is used for inversion calculation, to reflect the statistical law, each model is inverted 5 times, and the average value of the inversion results and the fitting error curve is taken as the final inversion result. The objective function of the inversion algorithm is calculated according to the Formula (11). Hankel transform, Gaussian integral, and Euler frequency-time conversion method were used for the forward solution. All algorithmic calculations were performed on a personal laptop with 32GB of memory and an Intel(R) Core(TM) i9-8950HK processor [46].

$$f=\sqrt{\frac{1}{N}\sum_{i=1}^{N}{\left[(h_{si}-h_{ci})/h_{si}\right]}^{2}}$$ (11)

where *N* is the number of time channels, *h*_*ci*_ is the forward calculation data, and *h*_*si*_ is the measured data.

### 3.1 Noise-free theoretical data analysis

**Fig 7:**
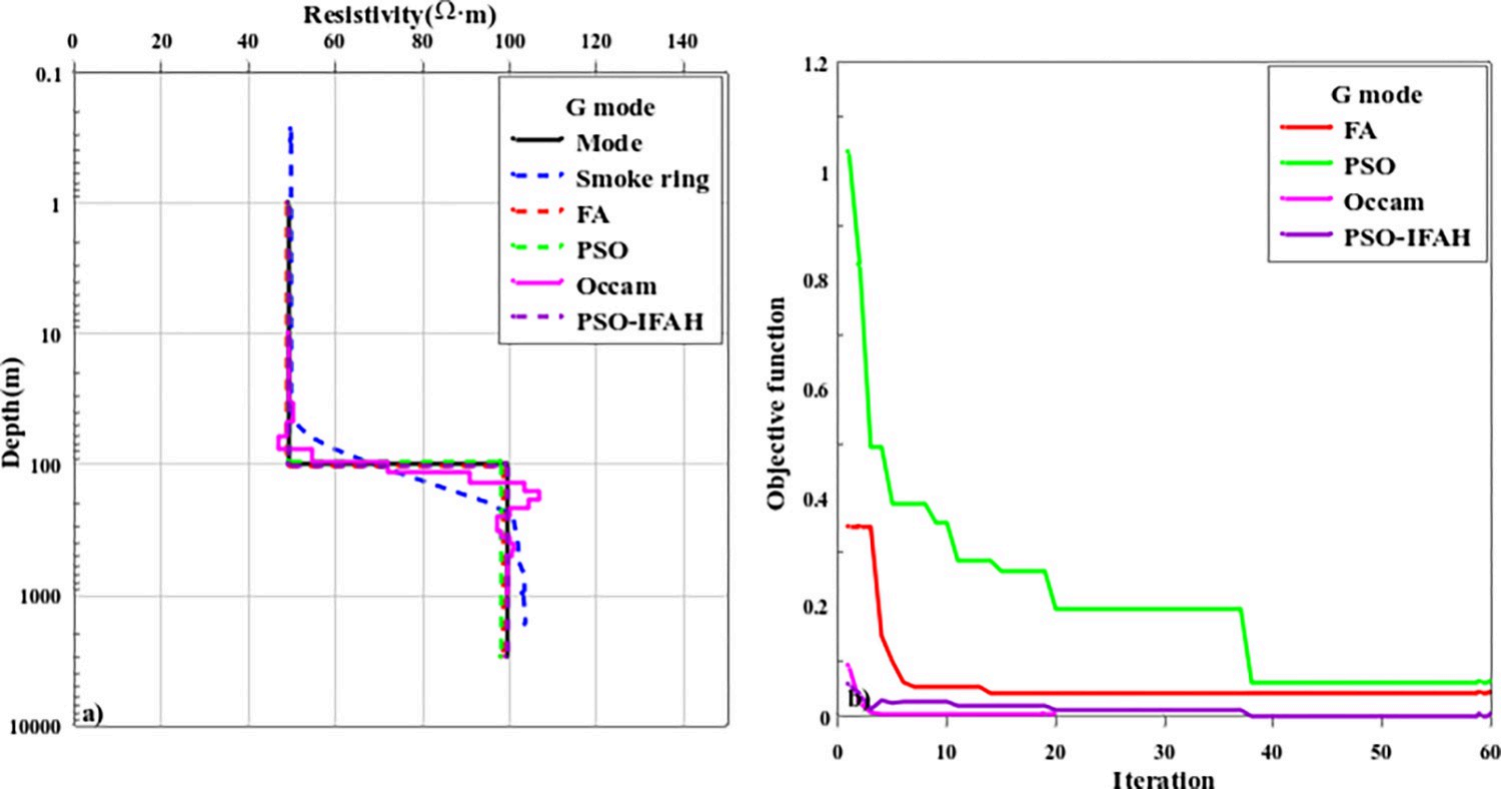
The inversion results and curve of the objective function of the G-type geoelectric model.

A two-layer G-type geoelectric model was established for analysis. Fig 7 shows the comparison curve of the results of five inversion methods of the two-layer G-type geoelectric model, respectively "smoke ring" fast imaging method, Occam inversion method, PSO algorithm, and FA algorithm, and improved PSO-IFAH optimization algorithm. In the G-type geoelectric model, the resistivity and layer thickness parameters are *ρ*_1_ = 50Ω·m, *ρ*_2_ = 100Ω·m; *h*_1_ = 100m, and the thickness of the second layer is infinite, respectively. As can be seen from the inversion results of the G-type geoelectric model in Fig 7(A), in terms of deep formation information, the inversion accuracy of the "smoke ring" fast imaging method is the lowest, followed by the Occam inversion method, the PSO and FA nonlinear optimization inversion methods have the highest accuracy, and the PSO-IFAH optimization algorithm has the highest accuracy. As can be seen from the curve of the change of objective function with the number of iterations in Fig 7(B), the inversion error of the PSO algorithm is the largest, followed by the inversion method of FA algorithm, and the inversion method of PSO-IFAH optimization algorithm and Occam is the smallest. In terms of algorithm computing efficiency, the PSO algorithm takes 5.12 min, the FA algorithm takes 14.83 min, and the PSO-IFAH optimization algorithm takes 6.98 min.

**Fig 8:**
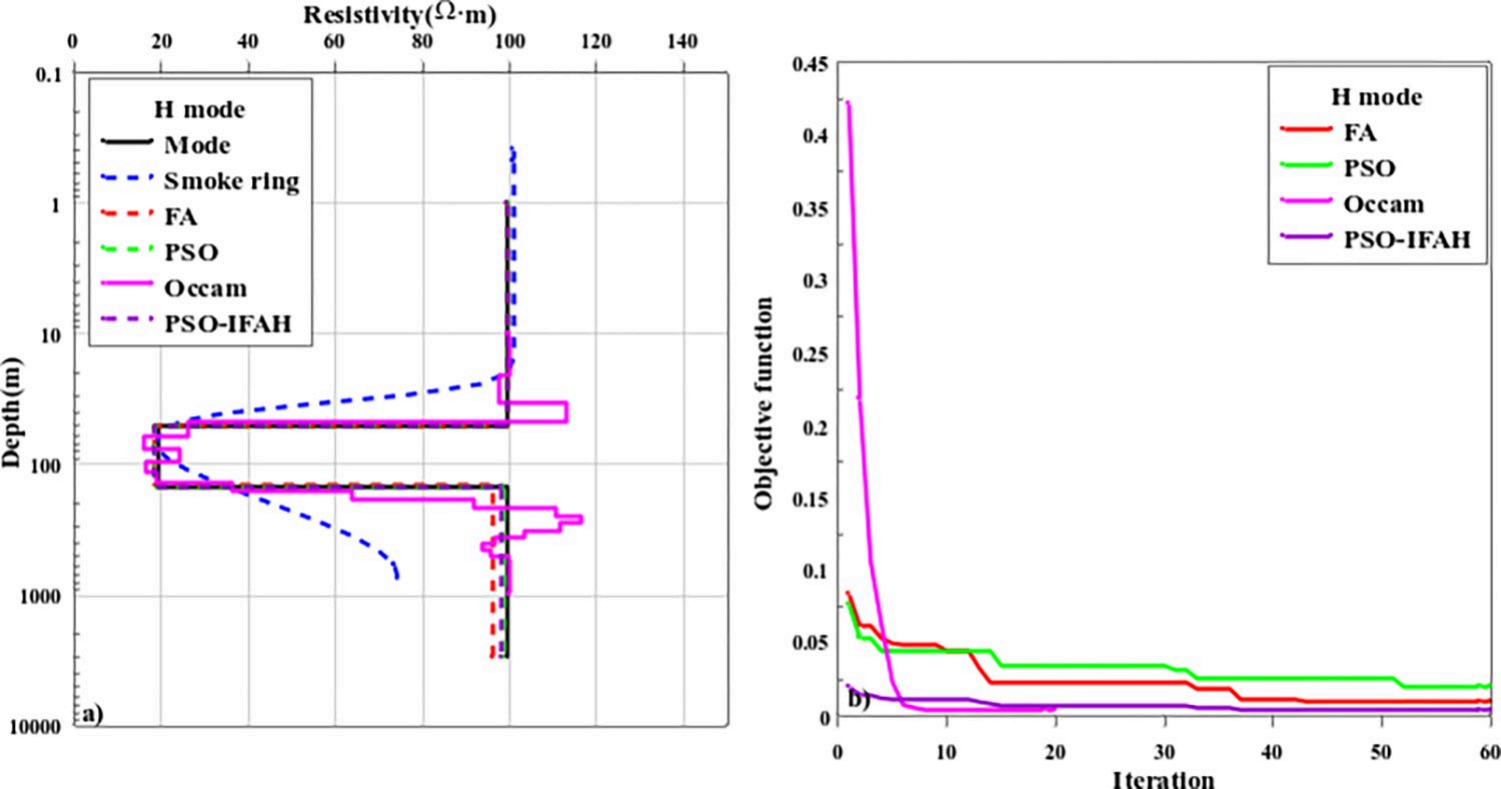
The inversion results and curve of the objective function of the H-type geoelectric model.

Fig 8 shows the H-type inversion results and objective function curves of the three-layer geoelectric model. In the H-type geoelectric model, the resistivity and layer thickness parameters of the layered dielectric model are *ρ*_1_ = 100Ω·m, *ρ*_2_ = 20Ω·m, *ρ*_3_ = 100Ω·m and the resistivity parameters show a high-low-high trend, *h*_1_ = 50m, *h*_2_ = 100m and the thickness of the third layer is infinite. In the process of nonlinear optimization inversion calculation, the initial model search range is *ρ*_1_∈[50,150], *ρ*_2_∈[1,50], *ρ*_3_∈[50,150], *h*_1_∈[10,100], *h*_2_∈[50,150]. As can be seen from Fig 8(A), in the H-type geoelectric model, the "smoke ring" rapid imaging method can reflect the information of the first layer of high resistance and the second layer of low resistance, but is not sensitive to the abnormal information of the third layer of high resistance. The error of the Occam inversion method is larger for the first and third layers with high resistivity, and smaller for the middle layer with low resistivity. PSO algorithm, FA algorithm, and PSO-IFAH optimization algorithm can accurately reflect the geoelectric parameters of the layered model. Due to the influence of the middle-low resistivity layer, the FA algorithm has errors in the inversion of the ground electric parameters of the third layer, and there is a certain deviation from the theoretical model. As can be seen from the change curve of the objective function with the number of iterations in Fig 8(B), in the first 10 iterations, the inversion error of Occam inversion method is the largest, followed by FA optimization inversion method, and PSO optimization inversion method is small. However, in the later iteration calculation, the error of the PSO optimization inversion method is greater than that of the FA inversion method. In the whole iterative calculation process, the PSO-IFAH optimization algorithm has the smallest error. In terms of algorithm calculation time, the Occam inversion method takes 1.17min, the PSO algorithm takes 6.72min, the FA algorithm takes 54.63min, and the PSO-IFAH optimization algorithm takes 7.87min.

**Fig 9:**
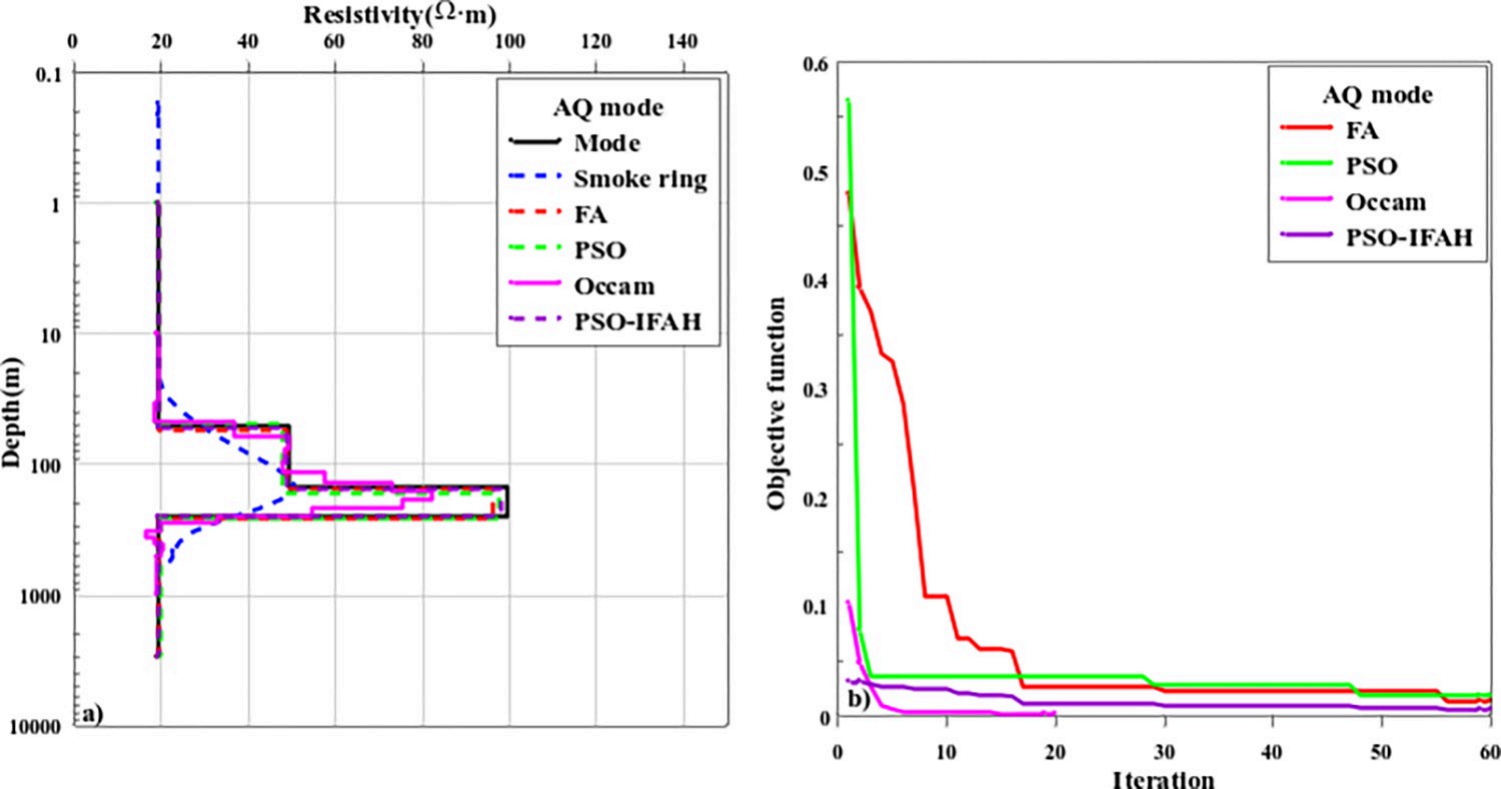
The inversion results and curve of the objective function of the AQ-type geoelectric model.

Fig 9 shows the AQ inversion calculation results and objective function change curves of the four-layer geoelectric model. In the AQ geoelectric model, the resistivity and layer thickness parameters of the layered dielectric model are *ρ*_1_ = 20Ω·m, *ρ*_2_ = 50Ω·m, *ρ*_3_ = 100Ω·m, *ρ*_4_ = 20Ω·m; *h*_1_ = 50m, *h*_2_ = 100m, *h*_3_ = 100m and the thickness of the fourth layer is set to infinity. In the process of nonlinear optimization inversion calculation, the initial search range of the AQ geoelectric model is as follows: *ρ*_1_∈[1,50], *ρ*_2_∈[10,100], *ρ*_3_∈[50,150], *ρ*_4_∈[1,50], *h*_1_∈[10,100], *h*_2_∈[50,150], *h*_3_∈[50,150]; As can be seen from Fig 9(A), the "smoke ring" rapid imaging method can only distinguish the parameter information of the first, second and fourth layers of the geoelectric model, while the third layer resistance anomaly is unable to be identified. Due to the influence of the fourth low resistance layer, the Occam inversion method has a weaker reflection on the third high resistance layer, and the error of inversion results is large. When the number of inversion layers increases, the PSO algorithm, FA algorithm, and PSO-IFAH optimization algorithm can effectively reflect the abnormal information of the formation. As can be seen from the change curve of the objective function with the number of iterations in Fig 9(B), the error of the PSO-IFAH optimization algorithm is smaller than that of the PSO algorithm and the FA algorithm. At the beginning of the iteration calculation, the error of the FA optimization inversion method is greater than that of the PSO optimization inversion method. However, with the increase in the number of iteration calculations, the error of the PSO optimization inversion method is gradually larger than that of the FA inversion method. In terms of algorithm calculation time, the Occam inversion method takes 1.15min, the PSO algorithm 7.63min, the FA algorithm 96.49min, and the PSO-IFAH optimization algorithm takes 9.21min.

**Fig 10:**
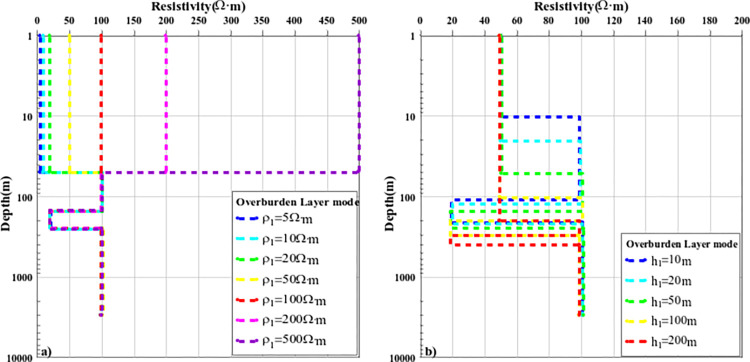
The curve of PSO-IFAH algorithm inversion results under the overburden layer model.

**Fig 11:**
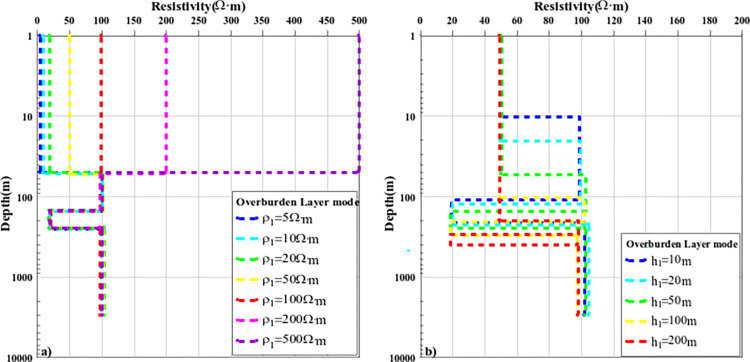
The curve of FA algorithm inversion results under the overburden layer model.

**Fig 12:**
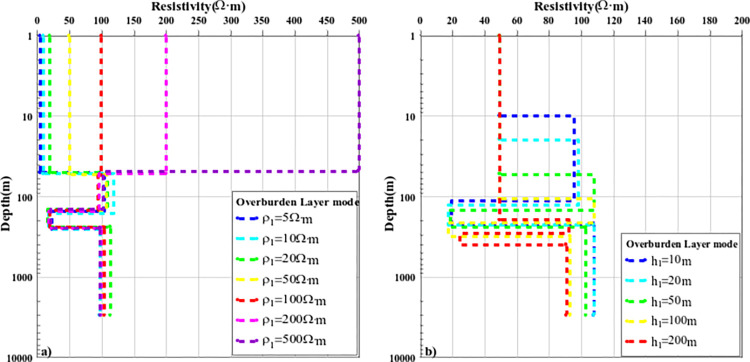
The curve of PSO algorithm inversion results under the overburden layer model.

The above content discusses the comparison results of the nonlinear optimization inversion methods of typical layered media models. To further analyze the effect of the improved PSO-IFAH algorithm, the covering layer model was established, and the calculation accuracy and inversion effect of the inversion method were discussed when the resistivity parameters and layer thickness of the overburden were different. A four-layer geoelectric model was established, and only one parameter of surface resistivity or layer thickness was changed each time, and the other parameters were fixed, and the inversion accuracy of each method was compared and analyzed. In the first case, the thickness of the fixed first layer is unchanged at 50m, and only the resistivity parameters of the surface layer are changed, and seven cases of 5Ω·m, 10Ω·m, 20Ω·m, 50Ω·m, 100Ω·m, 200Ω·m and 500Ω·m are selected for inversion calculation, and the inversion results are shown in Figs 10(A)–12(a); The inversion calculation is carried out in the five cases of 100m and 200m, and the inversion results are shown in Figs 10(B)–12(b). Fig 10 shows the inversion results of the PSO-IFAH nonlinear optimization algorithm under different resistivity and depth of the overlay layer. As can be seen from the figure, the PSO-IFAH algorithm can accurately and effectively reflect the surface resistivity information and has no impact on the inversion results of the deep formation anomaly information, and the inversion curves are consistent. Fig 11 shows the inversion results of the FA nonlinear optimization algorithm with different resistivity and depth of the overlay layer. It can be seen from the figure that when the surface resistivity gradually increases, the FA algorithm can accurately and effectively reflect the surface resistivity information and has no impact on the inversion results of the anomaly information in the deep formation, and the inversion curves are the same. As can be seen from Fig 11(B), when the thickness of the surface layer gradually increases, the inversion results of the FA algorithm are stable and reliable, which can effectively reflect the abnormal information of each layer. The disadvantage is that there is a certain error in the inversion of the resistivity parameters of the fourth layer, and the inversion curves are not completely consistent. Fig 12 shows the inversion results of the nonlinear optimization PSO algorithm with different resistivity and depth of the overlay layer. As can be seen from Fig 12(A), when the surface resistivity gradually increases, the PSO algorithm can effectively reflect the surface resistivity information, but it has an impact on the inversion results of the anomaly information in the deep formation, and there is a certain error with the parameters of the theoretical model, and the inversion curve does not coincide. As can be seen from Fig 12(B), when the thickness of the surface layer gradually increases, the inversion results of the PSO algorithm can reflect the abnormal information of each layer, but there is a large error in the inversion results of the resistivity parameters of the second and fourth layers. Therefore, in the case of different resistivity and layer thickness parameters of the overlay layer, the PSO-IFAH optimization inversion method has the best accuracy and stability in terms of algorithm inversion accuracy and stability, followed by the FA algorithm and the PSO algorithm.

### 3.2 Noise-contaminated theoretical data analysis

The characteristics of the optimization algorithm in terms of computational accuracy and stability are discussed above, and the results show that the improved PSO-IFAH optimization algorithm is better than other methods. Next, the characteristics of the improved PSO-IFAH optimization algorithm are further analyzed, and the anti-noise characteristics of the algorithm are discussed. The 5% and 10% Gaussian random noise were added to the forward data of the geoelectric model, and the PSO algorithm, FA algorithm, and PSO-IFAH optimization algorithm were used for inversion processing. The inversion results with the influence of noise are compared with the above noise-free theoretical inversion results, the relative error is calculated, and the anti-noise ability of the algorithm is analyzed.

**Fig 13:**
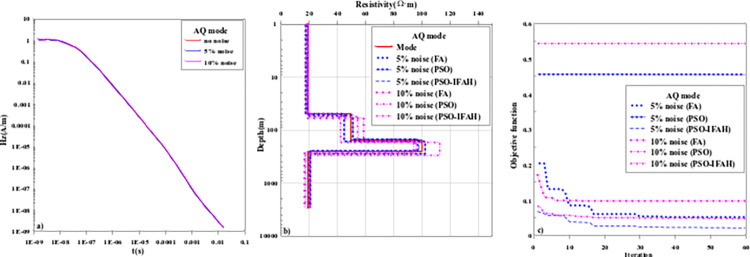
The inversion results and curve of the objective function of AQ-type geoelectric model with noise.

Fig 13 shows the inversion results and objective function curves of the AQ-type geoelectric model with noise, and the model parameters and inversion results are shown in Tables 1–3. It can be seen from Fig 13(B) that after adding random noise, the PSO algorithm and FA algorithm have large errors in the inversion results of the second and third layers of high resistivity anomalous information, and the inversion result curves deviate from the parameters of the theoretical model, while the inversion results of the first and fourth layers of low resistivity anomalies are better, and the inversion result curves are consistent with the theoretical model. As can be seen from Fig 13(C), the objective function error of the PSO algorithm is much larger than that of the FA algorithm and remains unchanged with the increase of the number of iterations, the objective function of the FA algorithm decreases with the increase of the number of iterations and gradually tends to be stable, while the error of the improved PSO-IFAH algorithm remains the smallest. It can be seen from Tables 1–3 that: (1) with the increase of inversion noise, the inversion error of PSO algorithm increases the most, followed by FA algorithm, and the improved PSO-IFAH algorithm has the smallest inversion error; (2) Regardless of the noise environment, the inversion error of the improved PSO-IFAH algorithm is smaller than that of PSO algorithm and FA algorithm, indicating that the improved algorithm has better anti-noise characteristics.

**Table 1:** The table of inversion results of AQ type geoelectric model with no noise.

Parameter	True value	Inverse results (PSO)	Relative error (%)	Inverse results (FA)	Relative error (%)	Inverse results (PSO-IFA)	Relative error (%)
ρ_1_(Ω·m)	20/Ω·m	19.99	0.05	20.26	1.30	20.05	0.25
ρ_2_(Ω·m)	50/Ω·m	48.62	2.76	49.65	0.68	50.22	0.44
ρ_3_(Ω·m)	100/Ω·m	98.17	1.83	98.35	1.65	99.37	0.63
ρ_4_(Ω·m)	20/Ω·m	20.66	3.32	20.12	0.64	20.01	0.05
h_1_(m)	50/m	49.17	1.66	50.48	0.97	50.12	0.24
h_2_(m)	100/m	115.71	15.71	96.32	3.68	97.61	2.39
h_3_(m)	100/m	97.74	2.26	102.60	2.60	101.30	1.30

**Table 2:** The table of inversion results of AQ type geoelectric model with 5% noise.

Parameter	True value	Inverse results (PSO)	Relative error (%)	Inverse results (FA)	Relative error (%)	Inverse results (PSO-IFA)	Relative error (%)
ρ_1_(Ω·m)	20/Ω·m	18.62	6.86	20.02	0.09	20.03	0.15
ρ_2_(Ω·m)	50/Ω·m	43.22	8.42	51.96	3.93	51.24	2.48
ρ_3_(Ω·m)	100/Ω·m	102.90	2.90	97.65	2.35	98.45	1.55
ρ_4_(Ω·m)	20/Ω·m	21.98	9.90	21.76	8.84	20.87	4.35
h_1_(m)	50/m	50.07	0.15	51.72	3.44	51.37	2.74
h_2_(m)	100/m	117.01	17.01	97.51	2.49	96.43	3.57
h_3_(m)	100/m	118.88	18.88	97.47	2.53	98.92	1.08

**Table 3:** The table of inversion result of AQ type geoelectric model with 10% noise.

Parameter	True value	Inverse results (PSO)	Relative error (%)	Inverse results (FA)	Relative error (%)	Inverse results (PSO-IFA)	Relative error (%)
ρ_1_(Ω·m)	20/Ω·m	18.46	7.68	20.02	0.11	20.06	0.30
ρ_2_(Ω·m)	50/Ω·m	43.22	13.55	59.52	19.04	52.46	4.92
ρ_3_(Ω·m)	100/Ω·m	113.13	13.13	94.75	5.24	97.62	2.38
ρ_4_(Ω·m)	20/Ω·m	21.49	7.48	19.81	0.91	21.63	8.15
h_1_(m)	50/m	49.84	0.31	51.01	2.02	51.63	3.26
h_2_(m)	100/m	124.29	24.29	108.11	8.11	95.87	4.13
h_3_(m)	100/m	130.12	30.12	110.12	10.12	96.54	3.46

## 4. Measured data analysis

The convergence, stability, and anti-noise ability of the improved PSO-IFAH algorithm are analyzed by theoretical forward analysis, and the results show that the algorithm has good performance. The following will be further analyzed through field measurements. The field measurement data comes from the ground transient electromagnetic karst survey of a subway line in a city, and the purpose of the survey is to detect the potential karst distribution under and around the tunnel. The geological conditions in the survey area are stable, the shallow surface is the Quaternary overburden, the middle and deep parts are composed of strong and weak weathered limestone, and the deep rock mass is relatively complete. The length of the transient electromagnetic field survey line is about 40m, and the point spacing is 2m. The PSO algorithm, FA algorithm, and improved PSO-IFAH algorithm were applied to the field measurement data, and the results of the "smoke ring" fast imaging method were demonstrated at the same time.

**Fig 14:**
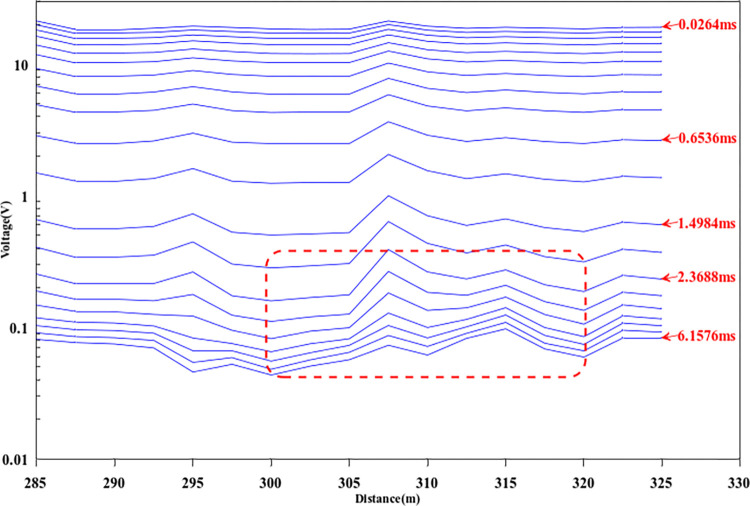
The primeval multi-channel map of the TEM method.

Fig 14 represents the original multi-track map of the transient electromagnetic field. As can be seen from the figure, the amplitude of the transient electromagnetic secondary field signal is enhanced between 2.3688ms and 6.1576ms between 300–320 m in the horizontal position, which is manifested as a low resistivity anomaly. According to the known geological data, the top-down stratigraphic sequence in the survey area consists of overburden, strong and weak weathered limestone in the middle, mixed clay and gravel layers, and intact limestone. Therefore, in the process of data inversion processing, based on the known geological information, an initial model is established for inversion calculation.

**Fig 15:**
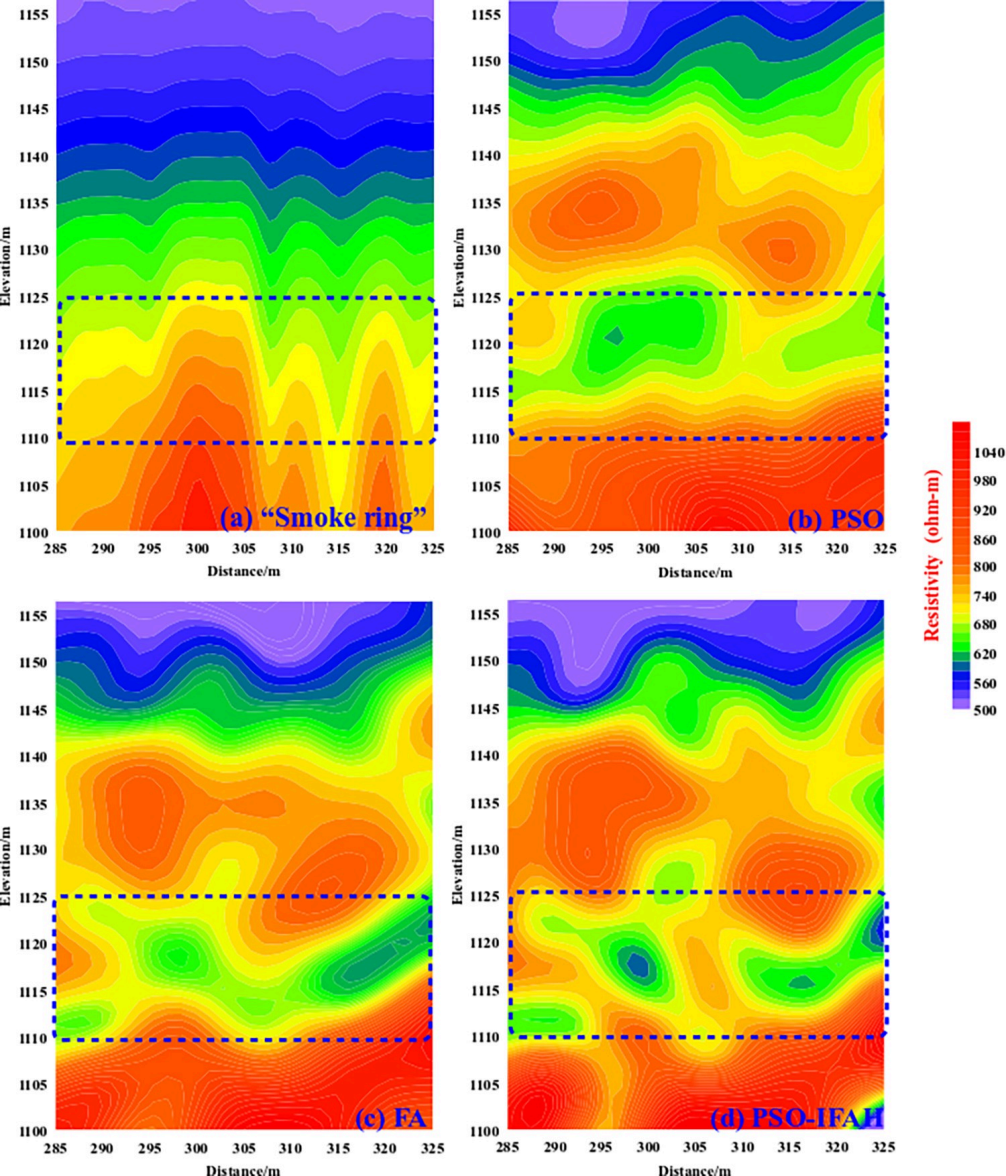
Comparison of inversion results for measured data.

Fig 15 shows a comparison of the four methods. The "smoke ring" fast imaging method is directly calculated, and the maximum number of iterations of the PSO, FA, and PSO-IFAH optimization inversion algorithms is set to 10 times in the nonlinear optimization inversion algorithm. According to the known geological data, the PSO algorithm, the FA algorithm, and the PSO-IFAH optimization inversion method data processing are calculated using the same search range and initial model. In terms of algorithm calculation time, the PSO-IFAH optimization inversion method is about 4.63 min, and the time consumption of the "smoke ring" fast imaging method is negligible. It can be seen from Fig 15(A) that the inversion results of the "smoke ring" fast imaging method show the shape of "hanging noodles", from Fig 15(B) and 15(C), it can be seen that the PSO algorithm and FA algorithm can reflect the subsurface low-resistance anomaly response between 295m and 320 m in the horizontal position and between 1110 and 1125m in depth, and it can be seen from Fig 15(D) that the PSO-IFAH optimization inversion method results have the high anomalous resolution, obvious low-resistance anomaly details, and high computational efficiency. Compared with the on-site excavation results, the inversion results of the improved PSO-IFAH optimization method are in good agreement with the actual situation.

## 5. Conclusions

The traditional algorithm for transient electromagnetic method inversion employs "smoke ring" fast imaging, which only reflects the approximate morphology of the stratigraphic model and has low precision. The nonlinear optimization inversion method provides a new solution for the fine inversion processing of TEM data. In this paper, the particle moving velocity of the FA algorithm is defined according to the concept of particle moving velocity in the PSO algorithm, and the appropriate velocity of particle movement is improved, so as to improve the local fast convergence ability of the FA algorithm and improved algorithm can overcome the oscillation problem around the optimal solution and improve the computational efficiency. Finally, an improved PSO-IFA hybrid optimization algorithm was proposed in the paper. The model tests with both theoretical and practical measurements demonstrated that the PSO-IFA hybrid optimization algorithm produces a high resolution and has good anti-noise properties and good convergence for the data. Processing the measurement data shows that the PSO-IFA hybrid optimization algorithm inversion results have more details than the PSO method and FA method. The research content provides a new and effective method for the fine data processing and interpretation of TEM.

However, the PSO-IFAH algorithm is currently only limited to one-dimensional inversion research. For high-dimensional inversion such as two-dimensional or three-dimensional inversion, the PSO-IFAH algorithm is not yet practical. Therefore, how to further solve high-dimensional inversion problems is the goal of our future research.

## Supporting information

S1 Data (RAR)
